# Supplementary material for: CXCL13 Positive Cells Localization Predict Response to Anti-PD-1/PD-L1 in Pulmonary Non-Small Cell Carcinoma
Source: Cancers (Basel). 2024 Feb 7;16(4):708. doi: 10.3390/cancers16040708 (PMC10887067; doi:10.3390/cancers16040708)
Supplement: Supplementary file 1 [file cancers-16-00708-s001.zip › cancers-2753861-supplementary.pdf]

Supplementary Tables and Figures:

Supplementary Table S1. Antibodies information for IHC experiments.

Supplementary Table S2. Clinicopathological characteristics of patients subdivided in PDL1-TPS groups.

Supplementary Table S3. Continuous variables of 65 NSCLC patients in our cohort.

Supplementary Table S4. Association between CXCL13+ cells in different compartments and OS

Supplementary Table S5. Association between CXCL13+ cells in different compartments and PFS

Supplementary Table S6. Association between other IHC markers and OS

Supplementary Table S7. Association between other IHC markers and PFS

Supplementary Table S8. Association between clinical features and OS

Supplementary Table S9. Association between clinical features and PFS

Supplementary Table S10. General multivariate COX models for all clinical and immunological (IHC markers) to predict OS.

Supplementary Table S11. General multivariate COX models for all clinical and immunological (IHC markers) to predict PFS.

Supplementary Table S12. Clinicopathological characteristics of the selected 65 NSCLC patients divided by TLS groups.

Supplementary Figure S1. All linear regressions between CXCL13+ cells in different compartments and OS.

Supplementary Figure S2. All linear regressions between CXCL13+ cells in different compartments and PFS.

Supplementary Figure S3. Correlation coefficients between CXCL13 in different compartments and other IHC markers.

Supplementary Figure S4. Comparing density of CXCL13+ cells in IM of the tumor in patients with no TLS with those with one TLS.

Supplementary Table S1. Antibodies information for IHC experiments.

	Antibody manufacturer	species	clone	dilution	incubation time	platform	HIER	positive control tissue
CD3	Dako-Agilent	rabbit polyclonal	N/A	pre-diluted	20	Autostainer 48 (DAKO)	High	tonsil
CD4	Dako-Agilent	mouse monoclonal	4B12	pre-diluted	20	Autostainer 48 (DAKO)	High	tonsil
CD8	Dako-Agilent	mouse monoclonal	C8/144B	pre-diluted	20	Autostainer 48 (DAKO)	High	tonsil
CD56	Dako-Agilent	mouse monoclonal	123C3	pre-diluted	20	Autostainer 48 (DAKO)	High	tonsil
CD163	Cell Marque	mouse monoclonal	MRQ-26	pre-diluted	32	Ventana Benchmark Ultra	CC1	tonsil
TIM3	Abcam	mouse monoclonal	EPR22241	1:1000	30	Autostainer 48 (DAKO)	High	lung adenocarcinoma
LAG3	Abcam	rabbit mono clonal	EPR20261	1:1000	20	Autostainer 48 (DAKO)	High	tonsil
FoxP3	Abcam	mouse monoclonal	236A/E7	1:500	15	Autostainer 48 (DAKO)	High	tonsil
PD-1	Abcam	mouse monoclonal	NAT105	1:100	20	Autostainer 48 (DAKO)	High	tonsil
CD20cy	Dako-Agilent	mouse monoclonal	L26	pre-diluted	20	Autostainer 48 (DAKO)	High	tonsil
CXCL13	R&D Systems	mouse monoclonal	53602	1:500	20	Autostainer 48 (DAKO)	High	tonsil

Supplementary Table S2. Clinicopathological characteristics of patients subdivided in PDL1-TPS groups.

Variables	PDL1-TPS < 1%	PDL1-TPS ≥1 and <50	PDL1-TPS > 50%
**Sex**			
Female	5	7	19
Male	6	9	11
**Smoking- habit**			
Former	8	10	24
Current	2	6	5
Never	0	0	2
**Histology groups**			
Adenocarcinoma	9	8	24
Squamous	1	5	4
**Ecog-status**			
0	4	6	7
1	7	10	19
2	1	0	2
3	0	0	2
**Stage of cancer at 1st immunotherapy**			
II	0	1	1
III	1	3	1
IV	10	12	28

Abbreviations: Ecog, Eastern Cooperative Oncology Group.

Supplementary Table S3. Continuous variables of 65 NSCLC patients in our cohort.

	Variable	Description	Median	Patients Above Median	Patients Below Median
0	Age	Continuous variable	68.3010	32	32
1	PDL1_TPS	Continuous variable	55.0000	16	28
2	CXCL13_T	Continuous variable	2.0000	32	29
3	CXCL13_IM	Continuous variable	0.5000	32	31
4	CXCL13_NT	Continuous variable	0.3000	25	24
5	CXCL13_TLS / CXCL13_T	Continuous variable	1.2840	31	31
6	CXCL13_T+IM	Continuous variable	2.0800	32	32
7	CXCL13_T+NT	Continuous variable	1.7610	26	26
8	CXCL13_T+IM+NT	Continuous variable	1.7315	26	26
9	CXCL13_TLSin	Continuous variable	0.0000	25	0
10	CXCL13_TLSne	Continuous variable	1.6270	31	31
11	CXCL13_TLS	Continuous variable	2.8630	31	31
12	CXCL13_TME	Continuous variable	2.3200	31	31
13	CXCL13_IM / CXCL13_T	Continuous variable	0.1860	30	30

14	CXCL13_IM / CXCL13_NT	Continuous variable	1.2665	21	21
15	TLS number	Continuous variable	0.0210	31	31
16	CD3	Continuous variable	49.94	32	32
17	CD4	Continuous variable	13.72	32	32
18	CD8	Continuous variable	7.49	32	32
19	CD56	Continuous variable	0.39	30	31
20	CD163	Continuous variable	12.60	32	32
21	PD1	Continuous variable	1.79	31	31
22	TIM3	Continuous variable	11.97	31	31
23	FoxP3	Continuous variable	1.02	31	31
24	LAG3	Continuous variable	0.39	30	30

Supplementary Table S4. Association between CXCL13+ cells in different compartments and OS. Univariate analysis of OS showed the high density of CXCL13 in sum of T, IM, NT as well as in NT increase the risk of death in NSCLC patients. T, tumor; IM, invasive margin; TLS, tertiary lymphoid structures; TLSin, inside TLS; TLSne, neighborhood of TLS; NT, non-tumor; TME, tumor microenvironment.

**The name of variable**	**Number of patients**	**HR**	**CI 95% lower**	**CI 95% upper**	**P-value***
CXCL13_T	65	1.09	0.99	1.21	0.09
CXCL13_IM	65	0.98	0.82	1.16	0.8
CXCL13_NT	52	1.13	1.02	1.26	**0.02**
CXCL13_T+IM	65	1.08	0.97	1.21	0.17
CXCL13_T+IM+NT	52	1.22	1.04	0.42	**0.01**
CXCL13_T+NT	52	1.23	1.07	1.42	**<0.001**
CXCL13_TLSin	63	1.00	0.97	1.03	0.78
CXCL13_TLSne	63	1.01	0.97	1.06	0.57
CXCL13_TLS	63	1.01	0.96	1.06	0.76
CXCL13_TME	63	1.07	0.97	1.18	0.16
CXCL13_TLS /CXCL13_T	63	0.83	0.69	1.01	0.06
CXCL13_IM / CXCL13_T	61	0.80	0.50	1.29	0.37
CXCL13_IM/CXCL13_NT	42	0.87	0.73	1.04	0.13

*Done with univariate COX regression. $p < 0.05$ marked in bold font shows statistical significance.

Supplementary Table S5. Association between CXCL13+ cells in different compartments and PFS. Univariate analysis of PFS showed the high density of CXCL13 in sum of T, IM, NT as well as in NT increase the risk of progression in NSCLC patients, while the high density of CXCL13 in TLS compared to the tumor decrease the risk of progression. T, tumor; IM, invasive margin; TLS, tertiary lymphoid structures; TLSin, inside TLS; TLSne, neighborhood of TLS; NT, non-tumor; TME, tumor microenvironment.

The name of variable	Number of patients	HR	CI 95% lower	CI 95% upper	P-value*
CXCL13_T	65	1.05	0.97	1.15	0.22
CXCL13_IM	65	1.06	0.95	1.18	0.31
CXCL13_NT	52	1.31	1.04	1.64	**0.02**
CXCL13_T+IM	65	1.06	0.97	1.16	0.18
CXCL13_T+IM+NT	52	1.16	1.02	1.32	**0.02**
CXCL13_T+NT	52	1.15	1.02	1.30	**0.02**
CXCL13_TLSin	63	1.00	0.97	1.03	0.93
CXCL13_TLSne	63	1.01	0.97	1.04	0.78
CXCL13_TLS	63	1.00	0.96	1.04	0.93
CXCL13_TME	63	1.05	0.97	1.13	0.27
CXCL13_TLS / CXCL13_T	63	0.84	0.73	0.98	**0.03**
CXCL13_IM / CXCL13_T	61	0.99	0.85	1.15	0.87
CXCL13_IM / CXCL13_NT	42	0.94	0.85	1.04	0.27

*Done with univariate COX regression. $p < 0.05$ marked in bold font shows statistical significance.

Supplementary Table S6. Association between other IHC markers and OS. Univariate analyses show that none of the other IHC markers can predict the risk of death in NSCLC patients.

The name of variable	Number of patients	HR	CI 95% lower	CI 95% upper	P-value*
CD3	65	0.99	0.97	1.01	0.45
CD4	65	1.01	0.97	1.05	0.61
CD8	65	0.97	0.92	1.02	0.25
CD56	63	0.71	0.25	2.02	0.52
CD163	65	0.98	0.92	1.04	0.50
TIM3	62	1.02	0.95	1.09	0.60
Lag3	61	0.01	0.00	3.54	0.12
FOXP3	63	1.08	0.83	1.40	0.59
PD-1	62	0.98	0.88	1.09	0.69

*Done with univariate COX regression.

Supplementary Table S7. Association between other IHC markers and PFS. Univariate analyses show that none of the other IHC markers can predict the risk of progression in NSCLC patients.

The name of variable	Number of patients	HR	CI 95% lower	CI 95% upper	P-value*
CD3	65	1.00	0.99	1.02	0.76
CD4	65	1.02	0.99	1.05	0.25
CD8	65	1.00	0.96	1.05	0.84
CD56	63	0.96	0.86	1.06	0.42
CD163	65	1.00	0.96	1.05	0.83
TIM3	62	1.00	0.95	1.05	0.87
Lag3	61	0.95	0.86	1.06	0.38
FOXP3	63	0.99	0.76	1.24	0.92
PD-1	62	0.98	0.91	1.06	0.66

*Done with univariate COX regression.

Supplementary Table S8. Association between clinical features and OS. Univariate analyses show that none of the clinical features can predict the risk of death in NSCLC patients. TPS, tumor proportion score; Ecog, Eastern Cooperative Oncology Group.

The name of variable	Number of patients	HR	CI 95% lower	CI 95% upper	P-value*
Age	65	0.96	0.92	1.01	0.09
Sex (Male vs Female)	65	1.08	0.52	2.24	0.8
PD-L1 TPS	57	0.99	0.98	1.00	0.21
Ecog-Status					
(1 vs 0)	59	1.12	0.41	3.03	0.82
(3,2,1 vs 0)	65	1.9	0.77	4.7	0.16
Histology group					
(Adeno vs Squamous)	60	1.26	0.53	2.96	0.6

*Done with univariate COX regression.

Supplementary Table S9. Association between clinical features and PFS. Univariate analyses show that none of the clinical features can predict the risk of progression in NSCLC patients. TPS, tumor proportion score; Ecog, Eastern Cooperative Oncology Group.

The name of variable	Number of patients	HR	CI 95% lower	CI 95% upper	P-value*
Age	65	0.99	0.95	1.02	0.44
Sex (Male vs Female)	65	1.14	0.6	2.03	0.65
PD-L1 TPS	56	1.00	0.99	1.01	0.64
Ecog-Status					
(1 vs 0)	59	1.00	0.34	2.6	0.94
(3, 2, 1 vs 0)	65	1.00	0.5	1.8	0.88
Histology group					
(Adeno vs Squamous)	60	1.41	0.7	2.84	0.33

*Done with univariate COX regression.

Supplementary Table S10. General multivariate COX models for all clinical and immunological (IHC markers) to predict OS. T, tumor; IM, invasive margin; TLS, tertiary lymphoid structures; TLSin, inside TLS; TLSne, neighborhood of TLS; NT, non-tumor; TPS, tumor proportion score; Ecog, Eastern Cooperative Oncology Group.

Overall Hazard Ratio:	Global P-value
0.71	0.5

The name of variable	Number of patients	HR	CI 95% lower	CI 95% upper	P-value*
CXCL13_IM	40	0.99	0.71	1.38	0.95
CXCL13_NT	40	1.03	0.76	1.39	0.85
CXCL13_TLSin	40	1.04	0.82	1.33	0.73
CXCL13_TLSne	40	1.20	0.40	3.6	0.75
CXCL13_TLS	40	0.80	0.21	3.06	0.75
CXCL13_T+IM+NT	40	1.07	0.64	1.79	0.78
CXCL13_TLS/CXCL13_T	40	0.96	0.48	1.94	0.91
CXCL13_T/ CXCL13_NT	40	1.01	0.90	1.15	0.82
CXCL13_IM/ CXCL13_T	40	0.99	0.71	1.38	0.95
CD3	40	1.00	0.94	1.06	0.96
CD4	40	1.02	0.94	1.11	0.65
CD8	40	1.01	0.91	1.11	0.89
CD163	40	0.98	0.85	1.15	0.84
PDL1-TPS	40	1.00	0.97	1.03	0.30
Age	40	1.00	0.91	1.09	0.97
Sex	40	0.74	0.11	5.22	0.77
Ecog-Status-1	40	1.07	0.23	4.97	0.93
Ecog-Status-2	40	0.95	0.08	10.72	0.97
Histology group	40	0.99	0.38	2.58	0.99

*Done with multivariate COX regression.

Supplementary Table S11. General multivariate COX models for all clinical and immunological (IHC markers) to predict PFS. T, tumor; IM, invasive margin; TLS, tertiary lymphoid structures; TLSin, inside TLS; TLSne, neighborhood of TLS; NT, non-tumor; TPS, tumor proportion score; Ecog, Eastern Cooperative Oncology Group.

**Overall Hazard Ratio:**	**Global P-value**
0.42	0.56

The name of variable	Number of patients	HR	CI 95% lower	CI 95% upper	P-value*
**CXCL13_IM**	40	1.00	0.76	1.31	0.97
**CXCL13_NT**	40	1.34	0.95	1.88	0.09
**CXCL13_TLSin**	40	1.10	0.87	1.39	0.42
**CXCL13_TLSne**	40	1.41	0.59	3.89	0.51
**CXCL13_TLS**	40	0.63	0.19	2.17	0.47
**CXCL13_T+IM+NT**	40	1.04	0.70	1.56	0.84
**CXCL13_TLS/CXCL13_T**	40	1.01	0.69	1.46	0.96
**CXCL13_T/CXCL13_NT**	40	1.05	0.94	1.18	0.37
**CXCL13_IM/CXCL13_T**	40	1.01	0.81	1.27	0.90
**CD3**	40	1.00	0.95	1.05	0.95
**CD4**	40	1.02	0.96	1.08	0.55
**CD8**	40	1.03	0.94	1.13	0.50
**CD163**	40	1.00	0.89	1.12	0.95
**PDL1-TPS**	40	0.99	0.97	1.02	0.67
**Age**	40	1.00	0.93	1.08	0.97
**Sex**	40	0.74	0.13	2.44	0.45
**Ecog-Status-1**	40	0.98	0.26	3.65	0.97
**Ecog-Status-2**	40	0.56	0.09	3.34	0.52
**Histology group**	40	0.75	0.34	1.67	0.48

*Done with multivariate COX regression.

Supplementary Table S12. Clinicopathological characteristics of the selected 65 NSCLC patients divided by TLS groups

The name of variable	No-TLS	Low-TLS	Intermediate-TLS	High-TLS
**Sex**				
Female	2	8	9	8
Male	9	5	4	3
**Smoking- habit**				
Former	10	10	11	5
Current	1	2	1	6
Never	0	1	1	0
**Histology groups**				
Adenocarcinoma	7	12	12	10
Squamous	4	1	0	2
**Ecog-status**				
0	4	8	4	2
1	6	4	8	7
2	0	1	1	1
3	0	0	0	1
**Stage at the start of immunotherapy**				
II	1	0	0	1
III	1	2	1	1
IV	8	11	12	9

Abbreviations: Ecog, Eastern Cooperative Oncology Group.

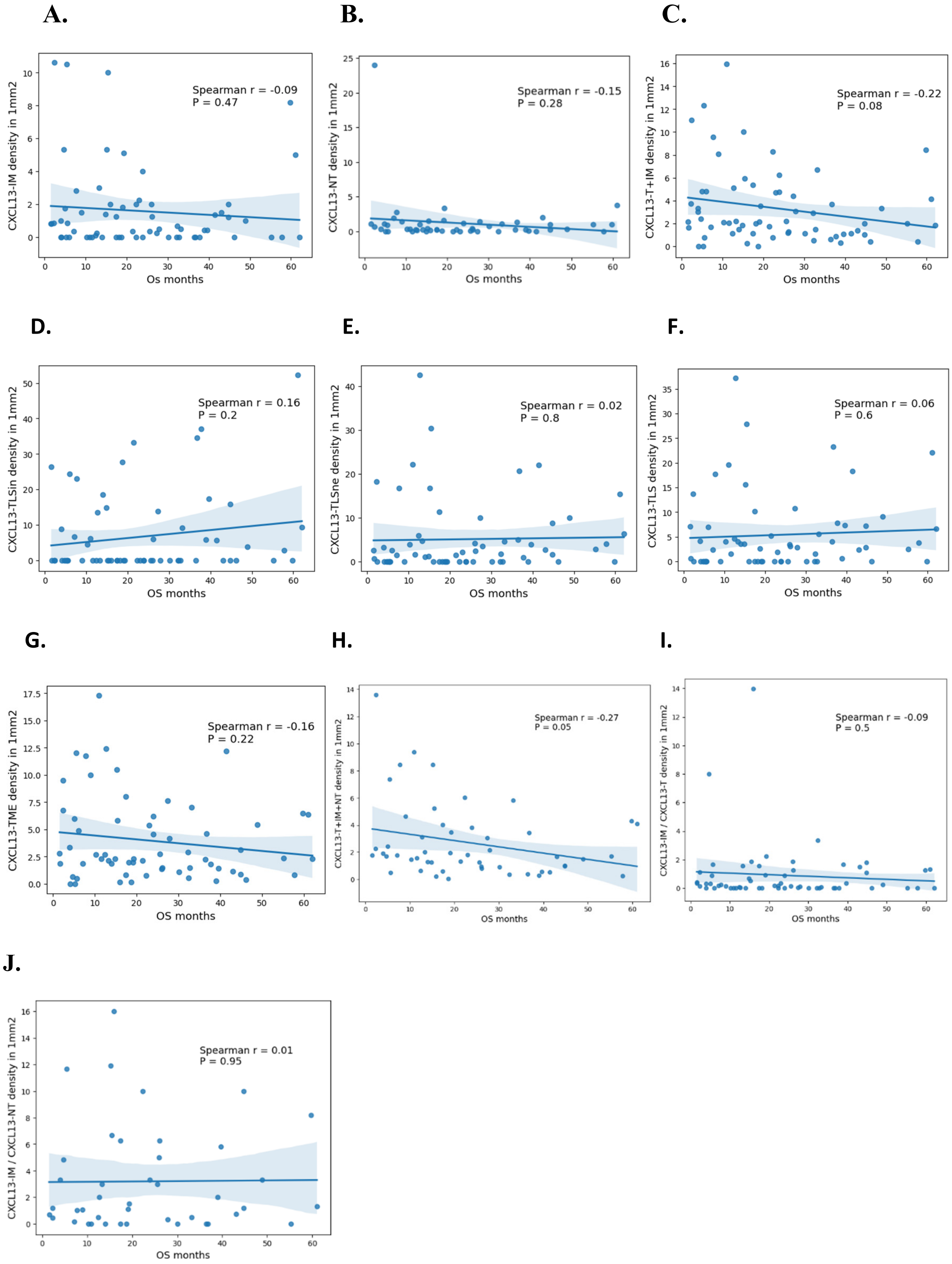
A.
CXCL13-IM density in 1mm2
Spearman r = -0.09
P = 0.47
Os months
B.
CXCL13-NT density in 1mm2
Spearman r = -0.15
P = 0.28
Os months
C.
CXCL13-T+IM density in 1mm2
Spearman r = -0.22
P = 0.08
Os months
D.
CXCL13-TLSin density in 1mm2
Spearman r = 0.16
P = 0.2
OS months
E.
CXCL13-TLSne density in 1mm2
Spearman r = 0.02
P = 0.8
OS months
F.
CXCL13-TLS density in 1mm2
Spearman r = 0.06
P = 0.6
OS months
G.
CXCL13-TME density in 1mm2
Spearman r = -0.16
P = 0.22
OS months
H.
CXCL13-T+IM+NT density in 1mm2
Spearman r = -0.27
P = 0.05
OS months
I.
CXCL13-IM / CXCL13-T density in 1mm2
Spearman r = -0.09
P = 0.5
OS months
J.
CXCL13-IM / CXCL13-NT density in 1mm2
Spearman r = 0.01
P = 0.95
OS months

Supplementary Figure S1. All linear regressions between CXCL13 in different compartments (IM, NT, T+IM, TLSin, TLSne, TLS, TME, T+IM+NT, IM/T, IM/NT) and OS. Correlation has asessed by Spearman's correlation analysis and linear regresion analysis. T, tumor; IM, invasive margin; TLS, tertiary lymphoid structures; TLSin, inside TLS; TLSne, neighborhood of TLS; NT, non-tumor; TME, tumor microenvironment.

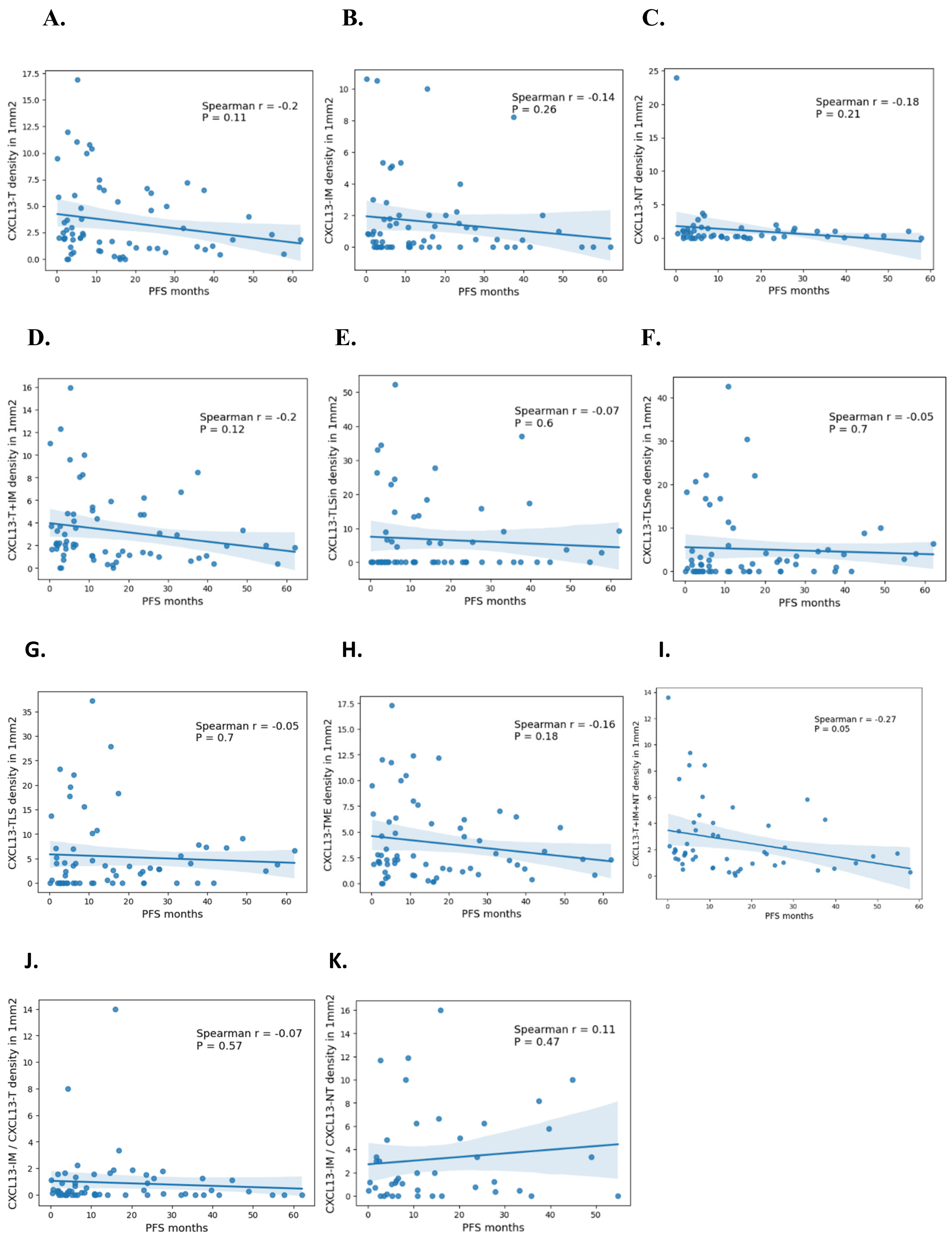

Supplementary Figure S2. All linear regressions between CXCL13 in different compartments (T, IM, NT, T+IM, TLSin, TLSne, TLS, TME, T+IM+NT, IM/T, IM/NT)

and PFS. Correlation has asessed by Spearman's correlation analysis and linear regresion analysis. T, tumor; IM, invasive margin; TLS, tertiary lymphoid structures; TLSin, inside TLS; TLSne, neighborhood of TLS; NT, non-tumor; TME, tumor microenvironment.

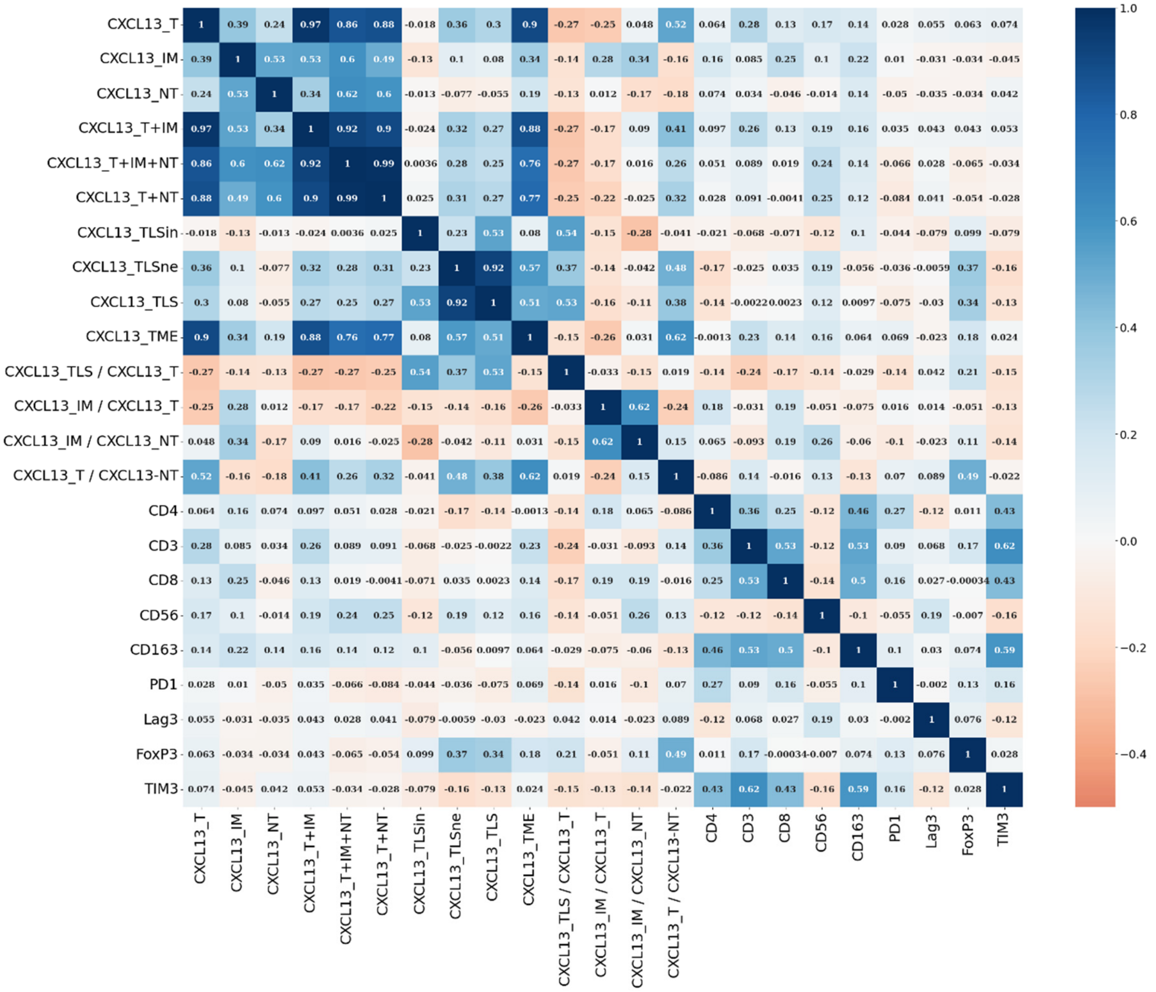

Supplementary Figure S3. Correlation coefficients between CXCL13 in different compartments and other IHC markers. The color scale and numbers within the heatmap represent the Spearman correlation coefficients. IHC markers are only given for the tumor area. T, tumor; IM, invasive margin; TLS, tertiary lymphoid structures; TLSin, inside TLS; TLSne, neighborhood of TLS; NT, non-tumor; TME, tumor microenvironment; Total, including tumor and stroma.

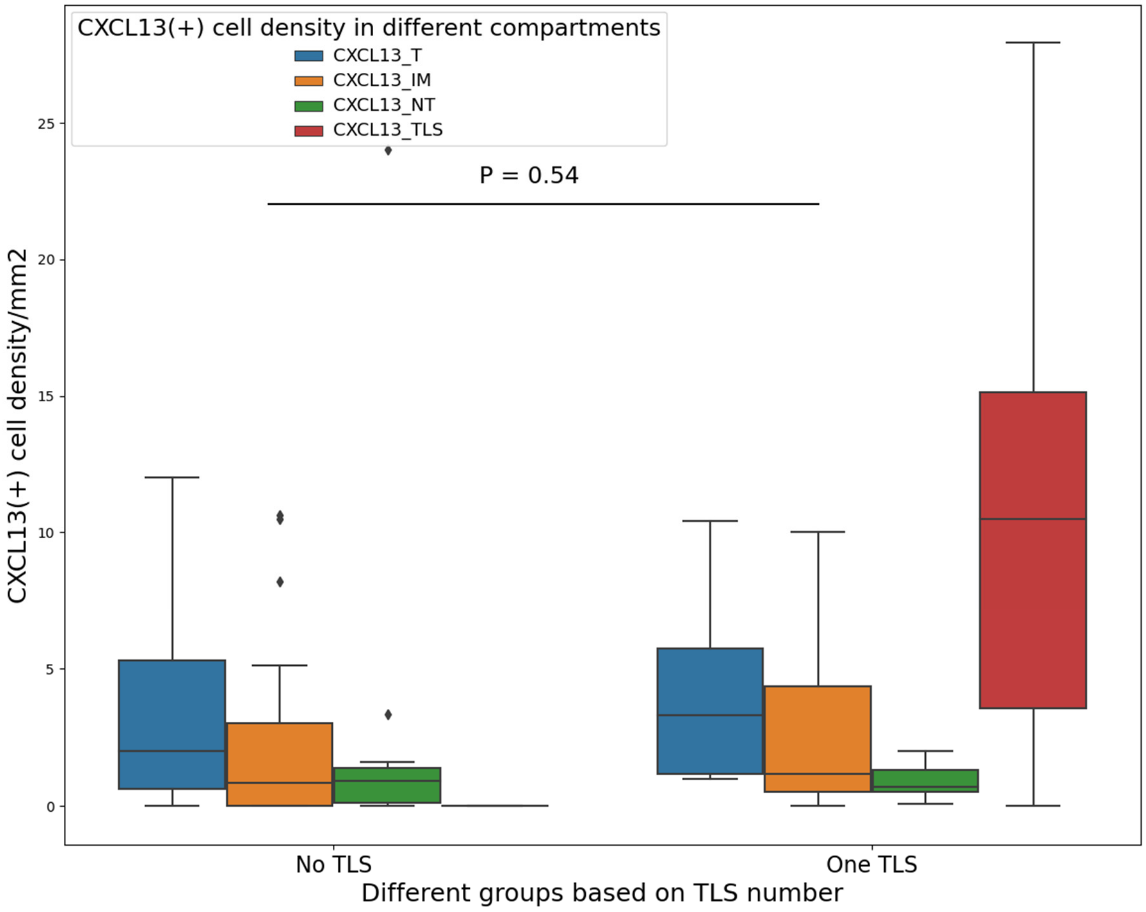

Supplementary Figure S4. Comparison of CXCL13+ cells density in patients with one or without TLS. T, tumor; IM, invasive margin; TLS, tertiary lymphoid structures; NT, non-tumor. *p*-value is given for the comparison between the absence and the presence of only one TLS groups for the IM compartment.
